# Correction: Smejda-Krzewicka et al. Interelastomer Reactions Occurring during the Cross-Linking of Hydrogenated Acrylonitrile-Butadiene (HNBR) and Chloroprene (CR) Rubbers Blends in the Presence of Silver(I) Oxide (Ag_2_O) and Mechanical Properties of Cured Products. *Materials* 2023, *16*, 4573

**DOI:** 10.3390/ma17051021

**Published:** 2024-02-23

**Authors:** Aleksandra Smejda-Krzewicka, Konrad Mrozowski, Krzysztof Strzelec

**Affiliations:** Institute of Polymer and Dye Technology, Lodz University of Technology, Stefanowskiego Street 16, 90-537 Lodz, Poland; 225373@edu.p.lodz.pl (K.M.); krzysztof.strzelec@p.lodz.pl (K.S.)

In the original publication [1], there was a mistake in Scheme 6 as published. There was a typing error. The carbon atom was incorrectly presented in three places. One carbon atom had a double bond instead of a single bond, while two other carbon atoms contained two hydrogen atoms instead of one hydrogen atom. The corrected Scheme 6 appears below. The authors state that the scientific conclusions are unaffected. This correction was approved by the Academic Editor. The original publication has also been updated.

## Figures and Tables

**Scheme 6:**
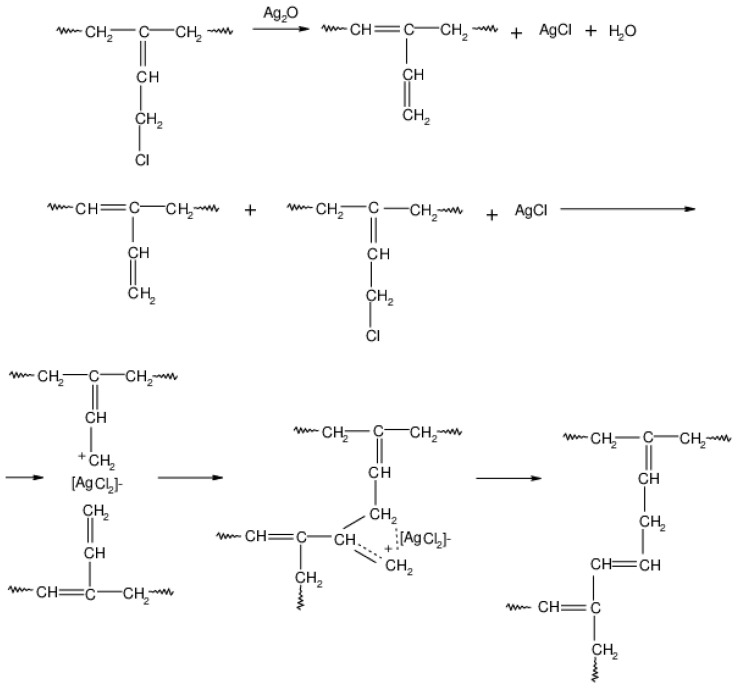
Cross-linking of chloroprene rubber (CR) in the presence of silver(I) oxide (Ag_2_O).
